# EMPOWERING FRONTLINE CNAS TO ENGAGE IN EFFECTIVE COMMUNICATION THROUGH AN INTERACTIVE LEARNING EXPERIENCE

**DOI:** 10.1093/geroni/igae098.0899

**Published:** 2024-12-31

**Authors:** Sarah Campbell, Neika Saville, Michael Bueno, Julie Rousseau, Ishan Varshney, Jung-Ah Lee, Lisa Gibbs

**Affiliations:** University of California Irvine Health, Irvine, California, United States; University of California Irvine Health, Irvine, California, United States; University of California Irvine, Irvine, California, United States; University of California Irvine, Irvine, California, United States; University of California Irvine Health, Irvine, California, United States; University of California Irvine, Irvine, California, United States; University of California Irvine, Irvine, California, United States

## Abstract

While considerable efforts have been directed towards continuing education for bedside nurses and providers, certified nursing assistants (CNAs) are often marginalized, despite their substantial involvement in patient care within skilled nursing and rehabilitation facilities. Collaborating with 36 sites throughout Orange County and Los Angeles County, California, our GWEP team identified crucial curricular topics to address geriatric-specific knowledge deficits among CNAs. These topics focused on the 4-Ms and included fall risk management, care for patients exhibiting challenging behaviors, addressing mental health conditions, effective communication with healthcare team members, and self-care strategies to mitigate burnout. Recognizing the importance of engaging and interactive education and trauma-informed pedagogy, we designed participatory activities such as gaming, storytelling, meditation, yoga, journaling, and sharing into the curriculum. Over a span of three years, education was delivered to over 615 diverse CNAs, nurses, and other healthcare professionals in our 4-part education series titled, “Building Resiliency While Advancing Your Caregiver Skills.” The curriculum was structured and disseminated through synchronous online sessions via Zoom, in-person workshops, and asynchronous self-paced modules. Throughout the sessions, empowerment and evidence-based burnout prevention were taught utilizing a customized “CNAs CAN” learning kit. Evaluations were conducted after each session, and we found that providing multi-modal learning opportunities promoted engagement. Knowledge was gained in reporting changes in condition, preventing falls, and recognizing the signs and symptoms of dementia, delirium, and depression. In the qualitative data collected, learners overwhelmingly reported gaining skills to prevent burnout, stress reduction, and acquiring new self-care skills.

